# Perceiving distance in virtual reality: theoretical insights from contemporary technologies

**DOI:** 10.1098/rstb.2021.0456

**Published:** 2023-01-30

**Authors:** Sarah H. Creem-Regehr, Jeanine K. Stefanucci, Bobby Bodenheimer

**Affiliations:** ^1^ Department of Psychology, University of Utah, Salt Lake City, UT 84112, USA; ^2^ Department of Computer Science, Vanderbilt University, Nashville, TN 37235, USA

**Keywords:** distance perception, virtual reality, visual cues

## Abstract

Decades of research have shown that absolute egocentric distance is underestimated in virtual environments (VEs) when compared with the real world. This finding has implications on the use of VEs for applications that require an accurate sense of absolute scale. Fortunately, this underperception of scale can be attenuated by several factors, making perception more similar to (but still not the same as) that of the real world. Here, we examine these factors as two categories: (i) experience inherent to the observer, and (ii) characteristics inherent to the display technology. We analyse how these factors influence the sources of information for absolute distance perception with the goal of understanding how the scale of virtual spaces is calibrated. We identify six types of cues that change with these approaches, contributing both to a theoretical understanding of depth perception in VEs and a call for future research that can benefit from changing technologies. This article is part of the theme issue ‘New approaches to 3D vision’.

## 1. Introduction

Virtual environments (VEs) are powerful tools that allow one to test theories of perception and spatial cognition [1,2]. They are useful because they allow for manipulation and control of environmental and body-based cues in ways that can be difficult or impossible in the real world. Yet the use of VEs for this purpose relies on the assumption that the perceptual information obtained in them is similar to what it would be in the real world, allowing for generalization of findings from one environment to another. A substantial body of work demonstrates that for the perception of absolute distance in VEs, though, there is a measurable and often large mismatch between perception in a VE and perception in the real world [3–5]. The scale (size and distance) of VEs is *underestimated* relative to real-world spaces—in some studies, distances have been underperceived by as much as 50%. Although the difference is less with newer equipment, it is still a significant issue, and the underlying perceptual causes of it are poorly understood.

This paper examines distance perception in VEs, with particular attention paid to results obtained with modern virtual reality equipment where we see improvement in the similarity of perception in VEs and the real world [5]. The goal is to describe the state of knowledge on distance perception in VEs with respect to which visual cues and other factors are missing, reduced, or altered by current technology, and then to explain why these cues and factors may affect distance perception. We focus on immersive VEs presented through head-mounted displays (HMDs) because the observed underestimation of distance perception is most prevalent in these devices. Further, we examine two main categories of factors that have been shown to *improve* distance perception in VEs. The first of these categories includes factors that are inherent to the observer and specifically to the observer’s visual and motor experiences in the VE. As we will discuss in further sections, research shows that visual-motor experience can reduce the underestimation of distance typically observed in VEs. For example, experience with a self-avatar as a visual cue in a VE [6] or locomotive experience in the VE [7] can re-scale the extent of the environment. The second category investigates factors inherent in the display technology (HMDs) themselves that may contribute to a reduction in underestimation of distance perception. For example, over a decade ago, research showed that the inertial properties (e.g. the weight) of an HMD affected distance perception [8], but as modern HMDs have become lighter in weight, distance perception has generally improved [5]. Overall, this paper will describe distinct areas in the study of distance perception in VEs that could shed light on visual cues that underlie calibration of the perception of scale in these environments: (i) visual experience; (ii) locomotor experience; (iii) body-based experience; (iv) weight of the HMD; and (v) field of view (FOV) of the HMD. For each factor, we discuss the research that suggests an effect on distance perception, the visual cues and other factors that may be reasons for *why* distance perception changes under the circumstances, and then open questions and future work that remain. We concede here that although our understanding of the reasons for why the perception of scale in VEs has improved compared to 20 years ago, we are still far from knowing all of them. This review will not be exhaustive in its coverage of all of the factors that could be involved. However, we hope that this paper will outline remaining challenges in certain areas that also can provide a call for more research to further understand the perception of scale in VEs going forward.

## 2. The problem of distance perception

How humans perceive the absolute scale of real environments, particularly distances over spaces that support locomotion, is a problem that has been posed by perceptual scientists for over half a century [9–16] and discussed by philosophers for much longer (e.g. [17]). Distance is perceived in absolute scale when it is defined with respect to a standard that is not part of the visual scene itself (e.g. metres or eye height), as opposed to relative relationships between spatial properties in the environment (e.g. object A is farther than B). Throughout the paper, we use the term *scale* to convey perceived absolute distance and size more generally; in other words, the sense of how big a space is perceived to be.

We focus on distance perception in the range of space beyond arm’s reach, termed *action space* by Cutting & Vishton [18]. This space allows for locomotion over relatively short distances up to about 30 m and has been substantially studied in VEs. We also focus on *egocentric* distance, or the perceived distance from the viewer to a target in depth (along the line of sight), as this is the perspective from which we naturally locomote and act. There are many cues for distance perception available to the visual system, but relatively few cues that specify absolute egocentric distance in spaces beyond around 3 m from the observer. Cutting & Vishton [18] provide an in-depth analysis of visual cues among different ‘zones’ of space (personal: up to 2 m, action: 2–30 m, and vista: beyond 30 m) which concludes that the *effectiveness of certain depth cues becomes attenuated with distance*. For example, accommodation (the change in the shape of the lens of the eye) and convergence (the angle between the optical axes of the eyes) are absolute distance cues that can be used to focus on objects at different distances, but are not effective beyond 2–3 m. At slightly farther distances, binocular disparity (the difference in the relative position of the projections of the same image on the two eyes) is primarily a relative depth cue, but can provide absolute information when combined with vergence angle. Motion parallax (the relative change in projections of objects caused by the viewer’s movement) is a powerful relative distance cue but can be an absolute cue to distance if the velocity of the viewer is understood and taken into account. Beyond oculomotor and motion cues, many monocular (pictorial) cues are effective within the zone of distances defined as action space, but only a few provide absolute depth information on their own and require assumptions to be met to determine absolute size and distance [19]. One example is familiar size (of objects or bodies), which could be used to determine distance if the object is assumed to be of typical size, by relating angular size to distance. Another example is eye height scaling of ground surface cues such as texture gradient and linear perspective. When an observer is standing on the ground, information about the height of the eye off the ground can be used to determine absolute distance to a location on the ground using the *angle of declination*, the angle between the line of sight to the horizon and the target on the ground.

The literature on perceived egocentric distances in action space in VEs commonly puts forward claims such as, *whereas distances are perceived accurately in the real world, they are underestimated in VEs.* Importantly, the notion of accuracy depends on the response measure used, even in the real world. Accurate egocentric distance perception in the real world is often demonstrated through a visually directed action task called *blind walking* in which an observer views a target and then walks without vision to its perceived location. On average, the distance walked is close to the actual distance, a finding interpreted as an accurate representation of absolute distance [11,20]. However, this accuracy in behaviour differs from verbal or numerical reports of distance given in standard metric units, which are often underestimated across both real and VEs [21,22]. There is some debate about whether this difference in accuracy is owing to the measure used, the information used, or interactions between them [16,23–26]. For example, some argue that the accuracy of blind walking should be understood as general systematic underestimation of perceived space (consistent with historic findings of foreshortening in depth (e.g. Wagner [27]) that is improved with visual-motor information from locomotion [16,23]. Others would claim that both measures access the same (accurate) perceived invariant location, but that verbal units are calibrated differently [22,24,25]. In all, there is general consensus that in the real-world egocentric distances are perceived as linear functions of physical distance regardless of the measure, but that underestimation is consistently seen in verbal reports [22]. In analysing the evidence for differences in distance perception between real and virtual spaces, we must recognize that our understanding of real-world distance perception depends on how accuracy is defined and on the measures used to assess perception. Warren [16] gives a helpful description of the paradoxes of distance perception and a summary of current theoretical accounts.

## 3. Factors that improve distance perception in virtual environments

Underestimation of egocentric distance (relative to intended distance or estimates in the real world) in VEs has been replicated over decades of research, although with a good amount of variation in magnitude (see Renner *et al.* [3] and Creem-Regehr *et al.* [4] for reviews). Early reports of action-based measures of egocentric distance perception were summarized in Thompson *et al.* [28] as 40–80% of actual distance. Further, Thompson *et al.* experimentally manipulated the level of the quality of graphics in an immersive VE and found that all levels showed about 50% underestimation in a triangulated walking task (walking without vision to a previously viewed target along an initial oblique path and then turning towards the target) as compared to near 100% accuracy in a matched real-world environment. Renner *et al.* [3] summarizes average egocentric distance estimation as 74% of the modelled distance, based on 78 articles published between 1993 and 2012. Kelly [5] found that the newest HMDs, on average, show distance estimation at about 82% of actual distance, which is improved, but still underestimated relative to the real world. Many factors have been examined as explanations for the underperception of scale including but not limited to: FOV and weight of HMDs [29–32], geometric distortions in displays [8,33–37], graphics quality or realism [28,38–40], pictorial or ground-surface cues [41–45], and response measures [38,46,47]. Although some of these variables influence distance estimates when manipulated, none have completely explained the differences between estimates made in VEs and the real world.

Despite the lack of a complete understanding of reasons for the underestimation, there is evidence that several factors have led to improvements in estimations of scale. These improvements are important for VE applications, but an understanding of *why* behaviour changes will facilitate both theoretical and applied approaches to the study of distance perception as well as provide guidelines for designers of VE applications that require accuracy. We group these factors by *experience: visual, locomotor, and body-based,* and *technology: HMD weight and FOV* to discuss their potential role in improving egocentric distance in the following sections.

### (a) Experience

#### (i) Visual experience

Experience with viewing the real-world prior to a VE improves perception of scale in VEs. For example, Interrante *et al.* [48] showed improved accuracy of egocentric distance estimates made in a VE that was a replica of the real world; however, the real world had to be seen first (see also an earlier study by Witmer & Sadowski [49] and a newer study by Feldstein *et al.* [50]). Studies subsequently showed that improved scaling of a real-world replica VE owing to viewing the real world first also transferred to a novel VE. These ‘transitional’ effects—gradually transitioning from a virtual replica of the real world to a different VE—were shown to improve both *presence* (the feeling of being in the environment) and the accuracy of distance estimation [51,52]. Effects of visual experience with an environment may also be bi-directional, with real world viewing influencing estimates in the VE and the VE influencing estimates in the real world [53]. The effects of visual experience may also be task specific; prior viewing of the real world improved blind walking estimates in a visually matched VE, but not size estimates [54]. These findings suggest that the improvement in distance perception gained from visual experience with the environment may not generalize to other aspects of scale (i.e. size perception).

Reasons for the effect of visual experience with the environment improving distance perception in VEs are not clear. VEs generally evoke more uncertainty about the scale of a space, which could be owing to many factors, including but not limited to differences in quality of the graphics in the VE, perception of eye height (discussed in the next sections), and the novelty of being in virtual reality itself. We hypothesize that any of these reasons for uncertainty in the scale of the space could lead to observers placing more weight on information about the context of the real-world environment when trying to estimate distance, thereby supporting an effect of visual experience of an environment on improvement in accuracy of distance estimation in VEs. The observer’s perception of space is naturally calibrated to the real world and visual experience could allow for transfer of this calibration to the VE. We revisit this concept of calibration (and the influencing factors) throughout this paper.

Future work could further examine the role of uncertainty about the scale or context of the VE and calibration provided by the real world by explicitly manipulating the match between real and virtual worlds. This is an approach that is possible with newer mixed-reality devices that allow for switching between a completely VE where traditionally the real world is not visible and an augmented reality (AR) environment where the real world is visually experienced (through optical see-through or camera-based devices) (see Jones *et al.* [55] for a similar approach with an older HMD). For example, viewers could be physically present in the real world viewing it through the HMD (as in AR) and then immediately experience the transition to a VE within the HMD that could match or not match the actual space to make distance judgements. If the specific context matters, then we might predict better performance in the matched versus mismatched VE context. If experiencing the real world through the HMD has more generalized effects on expectations of the scale of a space, then improved distance estimates might transfer to multiple environments, regardless of their match to the real world. A second approach is to examine distance estimation in AR itself, where virtual objects are presented as targets, but superimposed on the real-world space so that information for real-world context is inherent. In recent years, research on distance perception in AR has grown (see Kruijff *et al.* [56], Dey *et al.* [57], Erickson *et al.* [58] for reviews) but has yielded mixed results as to the accuracy of distance perception, probably owing to large variation in display characteristics [59].

#### (ii) Locomotor experience

Just as visual experience with a VE may serve to improve distance estimation, experience that provides continuous visual-motor feedback from walking through the environment also has an effect. Seminal studies by Rieser, Pick and colleagues [60,61] with a tractor-pulled treadmill in the real world showed that people learn and calibrate locomotion when given experience with new pairings of visual and biomechanical information for self-motion. The visual feedback is the rate of visual flow, or how quickly things in the environment pass by in central and peripheral vision as one moves. Biomechanical information is that gained from the body (e.g. proprioception, vestibular) as one walks. Early studies with locomotor experience in VEs showed that pairing ‘matched’ information from vision and biomechanics while walking with eyes open through the environment led to more accurate blind walked estimates of distance within a VE [62–64]. Further, people adjust their distance estimates based on other discrete feedback as well, such as visual or auditory feedback about their accuracy of blind walking that is given after they have completed the task, but this does not necessarily generalize to other response measures [62,65]. Finally, studies with mismatched visual and biomechanical information experienced during movement (e.g.manipulating the gain of visual information for self-motion so that it was faster or slower than speed of walking) led to *recalibration* of locomotor behaviour in the real world [62,66–70]. As in the completely real-world studies [60], manipulating gain in the VE changed the distance blind walked in the real world.

There are a number of possible explanations for the improvement in distance estimation shown after interacting with the VE. It could be that: (i) perceptual-motor recalibration has occurred owing to the experience of walking with visual feedback; (ii) perception of space has been re-scaled more generally owing to perceptual learning or differential weighting of cues; or (iii) perception itself has not changed, responses are explicitly corrected owing to a learned cognitive strategy. Testing the generalizability of feedback to different tasks has helped to compare these alternatives, although they are probably not mutually exclusive. If locomotion through a VE influences scaling only through perceptual-motor calibration then one might expect effects on distance estimates made with blind walking as the measure (owing to its reliance on this calibration), but not necessarily on other measures for distance perception that do not require action (e.g. verbal reports of distance). Alternatively, if experience with walking has a general effect on perceived distance or scale, then multiple measures would consistently reveal an effect of feedback on estimates. For example, given the tight coupling of perceived distance and perceived size [9,71], measuring size perception could test whether effects of feedback generalize across measures. Size judgements are also useful to rule out correction strategies because most participants do not have an explicit understanding of how perceived size changes with perceived distance [7]. Kelly *et al.* [7] assessed blind walking and size judgements in a VE and found that both improved after walking with continuous visual feedback, supporting the notion that re-scaling was generalized (but see Kunz *et al.* [69] who manipulated visual gain and did not find transfer to size estimates in the real world). Related work showed that experience within a specific zone of space for actions may also matter. Feedback for reaching did not influence blind walking or size judgments made in action space [7]. Also walking to objects 1–2 m away improved distance estimates for close distances but not farther distances (4–5 m) [72]. However, Siegel & Kelly [73] showed that improved blind walking and size estimates did generalize to distances farther than the space in which people interacted (1–5 m interaction generalized to 7–11 m distance as well as to size judgments). Further, similar effects on distance and size perception transferred to a different VE [74].

Taken together, this work suggests that experience with visual and locomotion feedback while moving through VEs can re-scale space perception within the VE in a way that does not seem to depend on only recalibration of walking or explicit conscious correction of responses. It may be that active interaction through a space gives more information for which cues reliably signal absolute depth [7]. For example, walking in the VE with eyes open not only provides experience with perceptual-motor coupling but also provides additional information about how angle of declination changes with distance to objects on the ground and how the sizes of texture elements change with distance. This scaling is well calibrated in the real world [16,23] but may not be initially within the VE. An open question is why sighted walking has a smaller effect on size judgments than on blind walking judgments [7,54]. One possibility is that blind walking relies on both updated perceptual-motor coupling (i.e. imagined updating of the environment as one moves) and a re-scaling of space, whereas non-action-based measures such as size are influenced only by the perceptual re-scaling. There is little known about how locomotion experience affects other non-action measures of VE scale such as verbal reports of distance (but see [62]) or perceived room size [75]. Some prior work using other experience-based manipulations such as varying realism of graphics [38,76], viewing the real world before a VE replica [54] or changing eye height [77,78] have found different results with different measures. This work suggests that there may not be one single factor that calibrates space [16,26], so further work is needed to determine how information for scale may be used differently for different task goals or response measures.

#### (iii) Body-based experience

**Figure 1.**
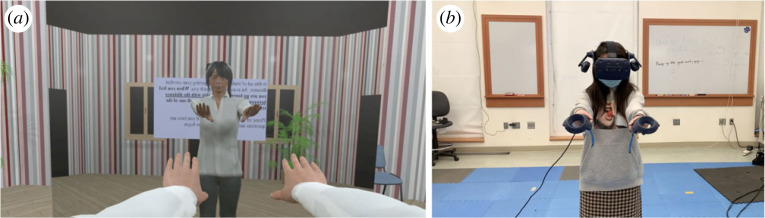
An avatar seen from a first-person and mirrored perspective (*a*) with tracked movements through hand-held controllers (*b*). (Online version in colour.)

Two cues inherent to the body size of the observer in a virtual environment could also lead to improvements in scaling of distance: self-avatars and information about eye height. Self-avatars, or graphical representations of the body that could be presented from either a first- or third-person perspective, provide useful information about the location of the observer in the space as well as the size of the observer (figure 1). They can also vary in visual aspects of presentation, such as being more human-like in appearance or more stylized, and can vary in terms of the extent of the body that is represented. For example, in prior work, sometimes just the participants’ feet and legs were represented in the virtual environment to provide cues for location and body size with which to scale distance or judgements for action [79–81]. In line with embodied theories of perception [26], self-avatars may also serve as a ‘ruler’ that can be used to better understand the scale of the virtual environment. Consistent with this account, viewing the world through a smaller or child’s body led to an overestimation of object size compared to a larger body [82,83]. Manipulating the size of a single effector, such as a hand or foot also has similar effects on perceived object size [84,85] and affordances [79], both of which convey information for scale.

One possible reason for the improvement in distance estimation could be the increased presence and calibration to the environment that a self-avatar provides when it is tracked to a user’s movements. Participants’ judgements of egocentric distance were more accurate in a VE when they were shown a full-body, self-avatar that was animated by their own movements [6,86] compared to a static avatar (but see also McManus *et al.* [87]). Gonzalez-Franco *et al.* [88] showed that people who reported higher embodiment with their avatar in an initial exploration session were better at blind walking in a subsequent distance estimation session. Kokkinara *et al.* [89] manipulated spatio-temporal properties of the body (so that visual feedback of arm motion was faster than actual movement) and found increased estimates of width and height of a box in a VE.

An increased sense of presence and the calibration of actions that occurs by viewing and moving an avatar are often confounded in experiments with avatars. In other words, viewing one’s moving avatar provides perceptual-motor feedback for scaling one’s actions in the VE but also greatly increases the sense of embodiment of the avatar (i.e. reporting that ‘I felt that I was the avatar’). More research is needed on both the type (e.g. visual, motor, full-body etc.) and amount of feedback (e.g. length of time, extent of natural movement) from avatars that is necessary to improve distance estimation. Much of the prior work on motor feedback provided from acting in VEs (as reviewed above) has focused on locomoting through space without the presence of an avatar. It is possible that new devices which track hands more naturally could lead to a quick re-scaling of the environment that would reduce the need for extensive experience with the environment.

**Figure 2.**
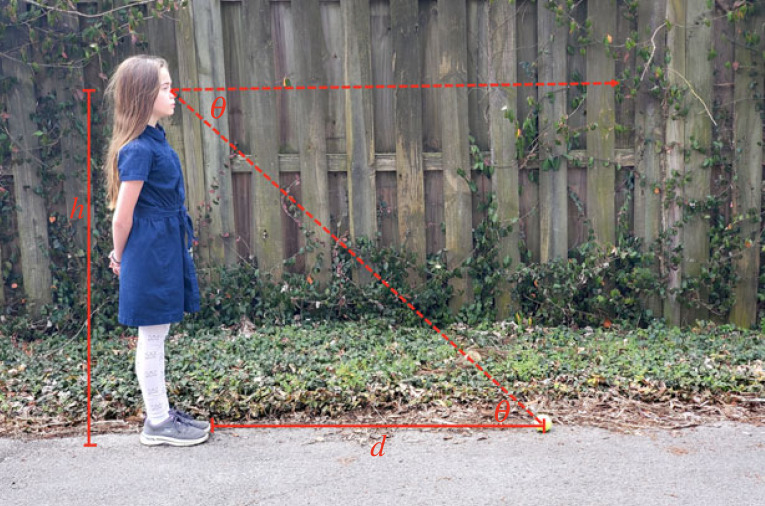
The egocentric distance *d* from an observer to a target resting on the ground is expressed in terms of eyeheight *h* by the formula d=hcot⁡θ, where *θ* is the angle of declination from the horizon. (Online version in colour.)

The second body-based cue for an observer that is highly important for perceiving scale in a VE is perceived eye height. The experience of one’s eye height in a VE is dependent on the tracking of the position of the HMD. Further, perceived eye height can also be dependent on the size and location of one’s self-avatar in a VE. How does eye height allow an observer to recover absolute egocentric distance? Sedgwick [12] defined the horizon-distance relation, more recently termed *angle of declination* as the mechanism. Here, he proposed that observers compute the distance to a target location on an infinite ground plane using the angle between the line of sight to the horizon and the line of sight to the target, scaled to the observer’s distance off the ground (or eye height), d=hcot⁡θ (figure 2). The horizontal line of sight may be determined by visual horizon information or by gravitational information (e.g. vestibular and proprioceptive feedback from the body). There is also evidence that when there is no ‘true’ visual horizon (as in a room where there is not an infinite ground plane), the floor-wall boundary serves as the visual horizon [90].

Many studies in the real world provide support for angle of declination as an important cue to egocentric distance. Ooi *et al.* [13] showed that increasing the angle of declination—manipulated by wearing prisms that induced a lowering of the target’s height in the field—led to underestimation of distance revealed through blind walking relative to a normal viewing condition (see also [21]). Minifying lenses also had a similar (but opposite effect), causing a decrease in angle of declination and an increase in perception of size [91]. Covertly manipulating eye height with false floors (creating a view of a floor that is different from the actual standing floor) [92] or raising the visual horizon [90] led to changes in size and distance perception in predicted directions.

Notably, it is much easier to manipulate eye height in VEs where visual and postural information can be decoupled [76] or the position of one’s self-avatar may present consistent or conflicting information [77,93]. Early work in VEs showed that lowering the visual horizon by truncating a richly textured ground plane (in an otherwise sparse environment) led to an increase in perceived distance relative to the normal horizon condition [94]. More recently, Leyrer and colleagues conducted an extensive set of experiments in VEs to examine how virtual eye height manipulations would affect perceived distance revealed by blind walking in both sparse and rich-cue environments [78,95], extending their earlier work that had used verbal reports [77]. They found that lowering eye height by 50 cm led to predicted increases in distance walked, but notably the magnitude of eye height change needed was large and less effective in the opposite direction (raising eye height did not differ from a matched eye height condition). Also, effects of lowered eye height were only observed when participants felt a strong sense of ownership of their self-avatar used to convey the extent of the eye height. Further supporting the role of eye height in distance perception in VEs, von Castell *et al.* [76] assessed verbal estimates of the dimensions of a room and showed that visual eye height was weighted more strongly than actual posture (by varying sitting and standing posture). They also found smaller effects in rooms modelled with more realistic textures and more context for inferring size, such as ceiling panels and windows. Other work has also shown better sensitivity to changes in eyeheight in realistic VEs with many familiar size cues [96].

Manipulations of eye height (and associated visual horizon and angle of declination) change distance estimates and could be a possible applied solution to counteracting distance underestimation effects. However, it is also notable that these manipulations do not necessarily match the level of magnitude of change in distance perception that is predicted by the trigonometric relationship between declination angle and eye height. They are probably most effective in the absence of other cues specifying scale. One such cue could be the presence of other objects in a realistic virtual rooms that can provide familiar size cues. More research is needed to test how manipulations of eye height interact with other information discussed in the current paper such as prior visual and motor experience. Furthermore, if angle of declination provides such a dominant cue to absolute distance, then it is important to ask how perceived eye height and angle of declination are influenced by HMD technology. We introduce and discuss this problem in the section on ‘Weight of the head-mounted display’ below.

### (b) Technology factors

#### (i) Weight of the head-mounted display

In the next two sections, we consider how changing HMD technology (which is associated with improved distance perception) could influence some of the information for absolute scale discussed in the earlier sections. First, the weight of the HMD has been directly [29,31] and indirectly [5] shown to affect distance perception. It is possible that wearing an HMD that increases weight on the head may affect how the *angle of declination* is computed or used. As described above, ample evidence from both real world and VE studies suggests that angle of declination is a strong cue for absolute size and distance [13,21,77,78,91,94]. In addition to vision, proprioceptive and vestibular cues provide information about eye height and head tilt. Although people are quite accurate in the real world at perceiving eye level or gaze along the horizontal [97], Durgin & Li [23,98] identified a bias in perceived *gaze declination* (overestimation by a factor of about 1.5) that is consistent with the phenomenon of underestimation of egocentric distance. Following the horizon-distance relationship described in figure 2, this increase in perceived angle of declination would lead to an underestimation of distance, which they propose as a unifying explanation for distance compression effects often demonstrated through verbal reports.

HMD weight could physically influence the posture and orientation of the head as well as perception of gaze declination. Thus, wearing an HMD may lead to an additional overestimation bias in angle of declination, explaining greater underestimation of distance in VEs when compared with the real world. Significant reductions in weight of the newer HMDs could also be an explanation for the attenuation of distance underperception. One study by Kuhl *et al.* [34] showed that pitching the entire virtual world up or down 5.7° around the eye point in an HMD did not influence blind walking judgments. This was a manipulation with respect to gravity that did not change the relative angle of declination specified with respect to the visual horizon (as in the prism manipulation in Ooi *et al.* [13]). The lack of effect on perceived distance observed in Kuhl *et al.* [34] suggests that people may prioritize their visual frame of reference more than body-based cues when faced with the uncertainty of VEs. However, this was only a single study with an older HMD and it may be that misperceptions of angle of declination owing to weight vary the magnitude of underestimation observed in VEs. Additional research is needed to assess whether HMDs of different weights cause different overestimation of perceived gaze declination using methods similar to Durgin & Li [98]. Further, direct manipulations of angle of declination in virtual reality could test whether correcting for potential effects of HMD weight can be accomplished outside of the technology.

#### (ii) Field of view of the head-mounted display

Another striking improvement in modern HMDs is the increase in their FOV. Although real-world restrictions of FOV do not greatly affect distance estimates ([29,30,33], but see [99]), manipulations of FOV within and across newer HMDs suggest that FOV is an important factor for distance perception in VEs [31,32,35,43,100–107]. Why would larger fields of view affect distance perception? One consequence of a larger FOV that more closely resembles natural vision in the real world is the increased visibility of the ground plane. Gibson [108, p. 6] argued strongly for the importance of the ground plane in his ground theory of perception, stating ‘there is literally no such thing as a perception of space without the perception of a continuous background surface’. Gibson’s early demonstrations illustrated that the perceived distance of an object is revealed partially through its perceived contact location with the ground. Texture gradients, regularly distributed patterns that change in scale with viewing distance, provide surface-related information for distance and size [12,108]. Studies on egocentric distance have shown that disrupting a uniformly textured ground surface between the viewer and target location reduces the accuracy of distance estimations [109]. Although the virtual ground can be seen with restricted vertical FOV in HMDs by scanning with head movements, this scanning makes integrating near ground surface cues that provide information for scaling farther distances more difficult and effortful [99]. In support of the importance of seeing the near ground surface, work has found that viewing the real world in the lower visual field of an HMD while viewing a matched virtual environment in the HMD improved distance estimation [110]. Jones *et al.* [111] also showed the importance of a larger vertical FOV for improved distance judgments. Recent work using virtual reality to simulate the very restricted FOV of typical AR devices found that affordance judgments for stepping over gaps on the ground were underestimated with a smaller FOV [112]. This underestimation of ability could have been owing to a misperception of the distance across the gap. Large, vertical FOVs also allow for seeing the environment close to the body, potentially providing more information about self-location and the ability to use scaling from the size of self-avatars when they are present. This idea has preliminary support from Nakano *et al.* [113], who modified an HMD with additional displays to have a vertical FOV of 130° (an increase of about 60°) and tested effects of the presence of a self-avatar on several subjective reports of experience. They found improvements in ratings of presence and sense of self-location with the combined larger vertical FOV and humanoid avatar, but no measures of distance perception were included in the study.

**Figure 3.**
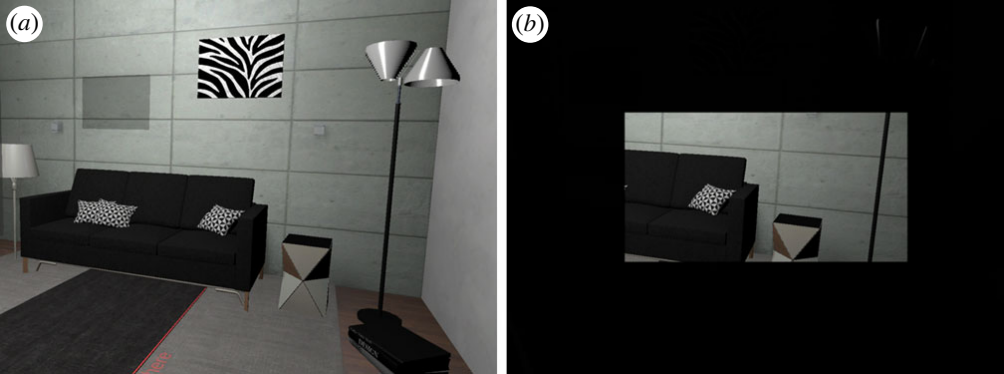
Reduced FOV of the HMD limits the environmental context that is visible without scanning (*b*) compared to the larger FOVs of contemporary HMDs (*a*). (Online version in colour.)

Studies motivated by calibrating the geometrical FOV (of the VE) with the HMD’s FOV showed that a mismatch between the two FOVs could lead to changes in perceived scale. For example, rendering the graphical imagery smaller than the display’s FOV, also called geometric minification, increases distance estimation [34,35,47,114]. This minification even led to overestimation of distances in the Oculus Rift (given near accurate performance with matched graphical and display FOVs). Minification affects a number of important cues for absolute distance perception including reducing the visual angle of objects—causing familiar size cues to signal farther distances—and reducing the angle of declination from the horizon to the target on the ground plane, which predicts an increase in perceived distance as well. However, in addition, minification increases the amount of environmental context provided in the periphery, consistent with the consequences of overall increased FOV (figure 3).

Effects of environmental context on distance perception, such as whether spaces are indoor versus outdoor, bounded versus open and small versus larger build environments, have been shown in both real and virtual worlds. For example, distances are estimated to be closer in outdoor versus indoor environments [21,43,115] and farther in bounded contexts (e.g. by the end of a hallway) than in unbounded contexts, such as an outdoor open space [116]. Recent work with virtual reality scenes presented on desktop displays found that increasing room width resulted in participants giving greater distance estimates compared to the same distance shown in a more narrow room [117]. With a series of experiments, Houck *et al.* [117] suggested that occlusion of either near or farther parts of the scene related to shorter distance estimations. However, this work was done with screen-based images, rather than in a HMD, so underestimation of distance could also have been owing to a misperception of eyeheight in the scene. Masnadi *et al.* [107] addressed environmental context and FOV in an extensive distance perception study, manipulating outdoor/indoor and cluttered/non-cluttered environments as well as both horizontal (165°, 110°, 45°) and vertical (110° and 35°) FOV within the same HMD. They found more accurate estimation in cluttered and indoor environments, as well as with increased horizontal FOV, supporting the importance of environmental context. However, no clear effects of manipulating vertical FOV were found in this study.

Wider horizontal and vertical FOVs are correlated with increased distance estimations, supporting the notion that FOV matters for scaling VE spaces. Larger FOVs increase visibility of ground surface and visual body-based cues, and reduce occlusion of the environment allowing for more environmental context. More work is needed to test for interactions among these cue types. Taking advantage of modern HMDs with larger FOVS and using the approaches of Masnadi *et al.* [107] and Nakano [113], both cues and effective FOV could be manipulated within the same device. For example, observers could experience the full wide FOV of the HMD and an artificially restricted FOV with either the presence or absence of their avatar body standing on the virtual ground while judging distances. Other approaches could examine the different types of wide and narrow environments like those used in Houck *et al.* [117], but with variation in HMD FOV to test whether environmental context manipulations have comparable effects to or interact with FOV manipulations.

## 4. Conclusion and future directions

**Figure 4.**
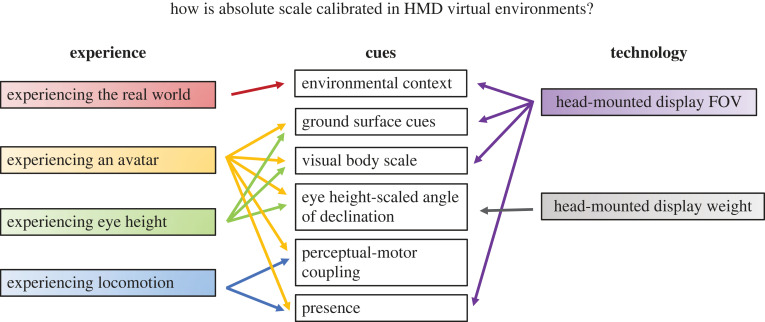
A model of how experience and technology influence six types of cues to calibrate the perception of absolute scale in virtual environments. (Online version in colour.)

We identified three *experience* factors that contribute to increased estimates of distance within the VE, thereby making perception of absolute scale more similar to that of the real world. These are: visual experience with the real-world environment, locomotor experience and body-based experience—further differentiated into experience with an avatar or eye height. In addition, two significant changes in HMD *technology* (FOV and weight) influence a subset of these cues. Our analysis suggests the need for additional calibration of scale in VEs and that different cues could provide this calibration. We summarize six types of calibration cues that are affected by experience and technology in figure 4. These are: environmental context, ground surface cues, visual body scale, eye height-scaled angle of declination, perceptual-motor coupling, and presence. While some of these cues relate directly to traditionally defined cues for absolute distance in the real world (e.g. ground surface cues or angle of declination) others are more specific to the unique circumstances of virtual worlds (e.g. visual body, perceptual-motor coupling, presence) where body-based information may need to be explicitly specified because of increased uncertainty owing to reduced or missing information.

Starting at the top of figure 4, our review points to *environmental context* as an influential cue both as a framework for grounding scale in a real-world context when there is uncertainty in the virtual world and to provide environmental features that structure perceived scale. Methodological decisions in VEs, such as allowing for *experience with the real world* before seeing its replica in a VE, could calibrate distance perception but this could be affected by the *FOV of HMDs* inherently affecting the availability of environmental features. FOV also affects the second category of cues—those of the *ground surface*—by limiting what can be seen without head movement. Decades of research in the real world has shown that the ground surface is critical for providing information for depth. Cues such as shadows, textures, and the horizon, provide relative information about depth. The observer’s body provides a way to scale that information to determine absolute distance and size. *Experiencing an avatar* and *experiencing eye height* in the virtual world affect that body scaling. Changing avatar body size and specified eye height (either tracking in a VE or visual cues) change both perception of *visual scale of the body* and *eye height scaled angle of declination*. *HMD FOV* also affects viewing the visual body and its location on the ground. It is also possible that the perceived angle of declination may be influenced by the *display weight*, consistent with prior work finding biases in perceived gaze angle and underestimation of distance. Beyond the more traditional spatial cues, we consider *perceptual-motor coupling* and *presence* in the calibration of distance. *Experiencing locomotion* provides a way to calibrate perception-action systems that appears to generalize beyond locomotion tasks to scale perceived space. It is difficult to separate out effects of perception-action calibration and presence, however, as the multisensory feedback gained from movement influences both.

The current studies and results presented here are promising for improving depth perception in VEs and for advancing our understanding of the underlying perceptual mechanisms by which improvement could occur. However, with recent advances in technologies and an analysis of the types of cues that may have the strongest effects, there are numerous future directions for research. For cues relating to *experience* of the user, new HMDs and tracking systems make it easier to implement body-based feedback through movement of avatar body parts, such as the hand. Given the strong effects of avatar bodies and locomotor experience on perceived scale, it would be useful to combine these approaches and test both factors together. Furthermore, much of the prior work with avatars used HMDs with FOVs that were more limited and required other methods for experiencing the body such as the use of virtual mirrors or movement training. Future work combining expanded FOV and avatars will be helpful for further understanding the role of more naturally accessible body-based cues. For other *technology* effects on cues, while the effects of overestimation of angle of declination have been established in the real world, it is unknown if this could explain increased (or relatively decreased) underestimation of distance with differently weighted HMDs. Direct manipulations of angle of declination in VEs would be a fruitful direction of future research as well.

Furthermore, there are other important characteristics of modern HMDs that are not currently presented here, as they have received little attention in research. These include the role of interpupillary distance (IPD)—the distance between the centres of the pupils of each eye—and display resolution or the number of pixels that can be displayed. IPD becomes an important issue as HMDs become more widely used across the lifespan, as most children (and many smaller adults) have IPDs that are smaller than the lowest setting in the device. Two studies have suggested that widening the IPD leads to an underestimation of distance [93] and size [37], but there are open questions about how these findings generalize across differently sized natural IPDs. Future research most relevant to improving applications should examine effects of mismatches between actual and device IPD on estimations of distance and size [36]. Effects of image quality owing to increased resolution (decreased pixel size) could also be an important area for future research given the role that real-world context and presence appear to play in calibration of scale. While there are few studies that directly examined this question, the abundance of new HMDs with ranges of resolution make this direction of research possible [5].

Finally, a question that continues to emerge across real world and VE investigations of distance perception is why some forms of experience affect some response measures but not others. Examining (and answering) this question will have important implications for theories of space perception and improving the use of virtual reality for applications. From a mechanistic perspective, recent proposals by Warren [16] and Proffitt [118] argue that there is not a singular calibrated perception of space and that certain tasks or goals may rely on different information (but see Philbeck *et al.* [25], Durgin [23], for arguments about unitary spatial representations). For applications that might depend on accuracy of perceived distance or size, it is important to understand when and why some manipulations of the cues associated with VE experience or technology generalize to different measures. Given the need to rely on observers’ responses to indicate their perception, this is ultimately a question about what it means to be calibrated to the absolute scale of VEs.

## Data Availability

This article has no additional data.
